# Tox2 is required for the maintenance of GC T_FH_ cells and the generation of memory T_FH_ cells

**DOI:** 10.1126/sciadv.abj1249

**Published:** 2021-10-08

**Authors:** Shu Horiuchi, Hanchih Wu, Wen-Chun Liu, Nathalie Schmitt, Jonathan Provot, Yang Liu, Salah-Eddine Bentebibel, Randy A. Albrecht, Michael Schotsaert, Christian V. Forst, Bin Zhang, Hideki Ueno

**Affiliations:** 1 Department of Microbiology, Icahn School of Medicine at Mount Sinai, New York, NY 10029, USA.; 2 Baylor Institute for Immunology Research, Baylor Research Institute, Dallas, TX 75204, USA.; 3 Global Health and Emerging Pathogens Institute, Icahn School of Medicine at Mount Sinai, New York, NY 10029, USA.; 4 Biomedical Translation Research Center, Academia Sinica, Taipei 11571, Taiwan.; 5 ImmunoConcEpT, CNRS UMR 5164, Bordeaux University, Bordeaux 33076, France.; 6 Genetics and Genomic Sciences, Icahn School of Medicine at Mount Sinai, New York, NY 10029, USA.; 7 Department of Immunology, Graduate School of Medicine, Kyoto University, Kyoto 606-8501, Japan.; 8 Institute for the Advanced Study of Human Biology, Kyoto University, Sakyo-ku, Kyoto 606-8501, Japan.

## Abstract

Memory T follicular helper (T_FH_) cells play an essential role to induce secondary antibody response by providing help to memory and naïve B cells. Here, we show that the transcription factor Tox2 is vital for the maintenance of T_FH_ cells in germinal centers (GCs) and the generation of memory T_FH_ cells. High Tox2 expression was almost exclusive to GC T_FH_ cells among human tonsillar and blood CD4^+^ T cell subsets. Tox2 overexpression maintained the expression of T_FH_-associated genes in T cell receptor–stimulated human GC T_FH_ cells and inhibited their spontaneous conversion into T_H_1-like cells. Tox2-deficient mice displayed impaired secondary T_FH_ cell expansion upon reimmunization with an antigen and upon secondary infection with a heterologous influenza virus. Collectively, our study shows that Tox2 is highly integrated into establishment of durable GC T_FH_ cell responses and development of memory T_FH_ cells in mice and humans.

## Teaser

Transcription factor Tox2 plays a key role to maintain T_FH_ phenotype and generate memory T_FH_ cells in both mice and humans.

## INTRODUCTION

Immunological memory is fundamental to protect the host from a reinfection of the pathogen. The maintenance of humoral memory is mediated by long-lived plasma cells and memory B cells (*1*). Their development requires helper signals, including CD40, provided by T follicular helper (T_FH_) cells in germinal centers (GCs) (*2*–*4*). GC T_FH_ cells participate in the selection of high-affinity B cells and their differentiation into long-lived plasma cells and memory B cells (*5*–*7*) and therefore are fundamental for the generation of durable humoral responses.

The existence of memory T_FH_ cells has been demonstrated in mice, nonhuman primates, and humans (*8*–*11*). Memory T_FH_ cells play essential roles in the secondary antibody (Ab) response and provide help to memory B cells and naïve B cells. Human blood circulating T_FH_ cells (cT_FH_) efficiently provide help to B cells in vitro via the expression of CD40L as well as cytokines such as interleukin-21 (IL-21), IL-10, and IL-4 (*11*–*16*). Memory T_FH_ cells also remain in lymphoid organs as local memory T_FH_ cells (*17*), and current evidence shows that memory T_FH_ cells can be derived from mature GC T_FH_ cells and T cells committed to the T_FH_ lineage yet not fully mature. The latter pathway is evident by the observation that animals deficient of functional stress-activated protein [SLAM-associated protein (SAP)], which lack the development of GC T_FH_ cells, can still generate memory cT_FH_ cells (*16*). Memory T_FH_ cells can persist in mice without the presence of the antigen (*9*, *16*, *18*). In humans, vaccinia virus–specific cT_FH_ cells can remain for decades after the vaccination (*19*), an observation indicating their persistence without antigens. The molecular mechanism required for the initial development of memory T_FH_ cells largely remains unclear.

Tox2 is a transcription factor that belongs to the Tox (thymocyte selection–associated HMG box) family, which is composed of Tox1, Tox2, Tox3, and Tox4 (*20*). Here, we show evidence that the transcription factor Tox2 is required for the maintenance of T_FH_ cells in both humans and mice. Tox2 overexpression maintained the expression of T_FH_-associated genes in T cell receptor (TCR)–stimulated human GC T_FH_ cells and inhibited their spontaneous conversion into T helper 1 (T_H_1)–like cells. Tox2-deficient mice displayed impaired secondary T_FH_ cell expansion upon reimmunization with an antigen and upon secondary infection with a heterologous influenza virus. Tox2-deficient mice also increased immunoglobulin G2b (IgG2b) and IgG2c production with increased interferon-γ (IFN-γ) production after primary immune response. Our study shows that Tox2 is highly integrated into durable GC T_FH_ cell response and development of memory T_FH_ cells in mice and humans.

## RESULTS

### Human GC T_FH_ cells highly express Tox2

**Fig. 1.**
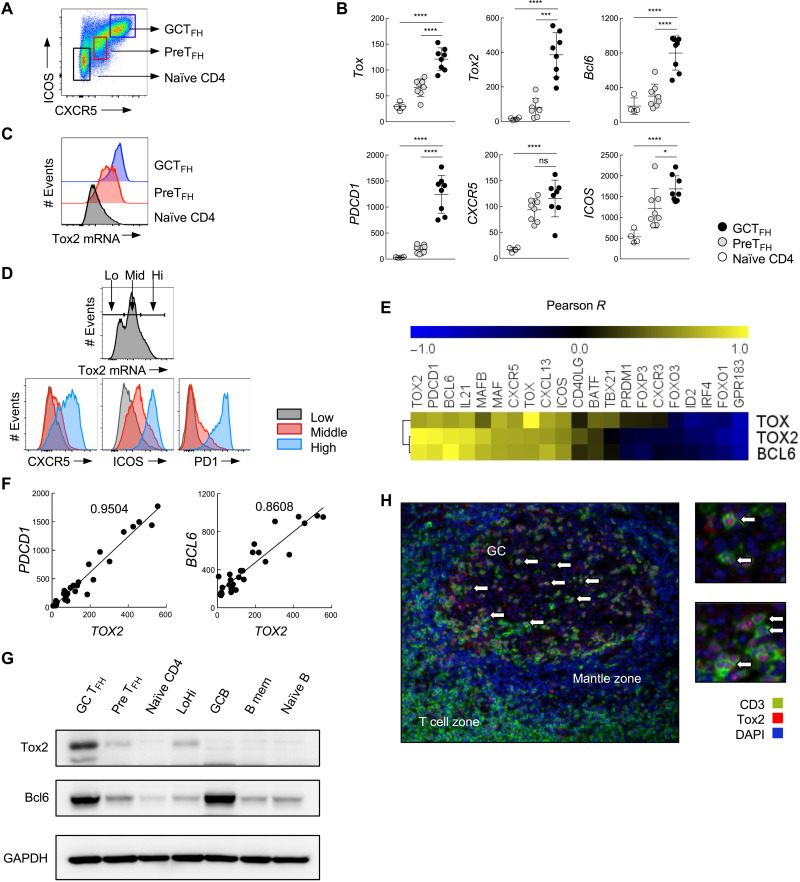
Tonsillar GC T_FH_ cells express abundant Tox2 and positively correlates with BCL6 and PDCD1. (**A**) Three tonsillar CD4^+^ T cell populations were defined according to the expression of ICOS and CXCR5: CXCR5^hi^ICOS^hi^ as GC T_FH_, CXCR5^lo^ICOS^lo^ as PreT_FH_, and CXCR5^−^ICOS^−^ as naïve CD4. (**B**) mRNA expression of *TOX*, *TOX2*, and T_FH_-related genes assessed by NanoString in human tonsillar CD4^+^ T cell subsets. *P < 0.05, ***P < 0.005, ****P < 0.001. (**C**) Flow RNA analysis of *TOX2* in human tonsillar CD4^+^ T cell subsets. (**D**) Flow RNA analysis of *TOX2* and its association with T_FH_ molecules in human tonsillar CD4^+^ T cells. (**E**) Correlation between *TOX2*, *TOX*, and *BCL6* mRNA and T_FH_-related molecule mRNA, indicated by Pearson *R* values. (**F**) Correlation of *TOX2* mRNA with *PDCD1* and *BCL6* mRNA in tonsillar CD4^+^ T cell populations. Pearson *R* values are shown. (**G**) Expression of Tox2 and Bcl6 proteins in tonsillar CD4^+^ T cell populations and B cell populations was assessed by Western blotting. Equal amounts of protein were loaded. (**H**) Localization of Tox2-expressing cells (red) and T cells (green) was analyzed by immunohistochemistry using a frozen tonsil section. White arrow indicates Tox2^+^ T_FH_ cells in GCs. ns, not significant.

The comprehensive transcript profiles of human blood and tonsillar CD4^+^ T cell subsets CXCR5^hi^ICOS^hi^ (GC T_FH_), CXCR5^lo^ICOS^lo^ (T_FH_ precursors: PreT_FH_), and CXCR5^−^ICOS^−^ (naïve) CD4^+^ T cells (Fig. 1A) were analyzed by mRNA microarray (fig. S1A). High expression of *TOX2* was almost exclusive to tonsillar PreT_FH_ and GC T_FH_ cells, and GC T_FH_ cells were the highest (fig. S1A). A 2-day activation with CD3-CD28 monoclonal Abs (mAbs) did not up-regulate *TOX2* by blood CD4^+^ T cell subsets (fig. S1A), indicating that high *TOX2* expression by GC T_FH_ cells was not due to the difference in activation status. High expression of *TOX2* by GC T_FH_ cells was confirmed by NanoString (Fig. 1B). The analysis by flow cytometry with in situ mRNA hybridization assay revealed that GC T_FH_ cells universally expressed high levels of *TOX2* (Fig. 1C). The expression of *TOX2* in tonsillar CD4^+^ T cell subsets showed a positive correlation with CXCR5, ICOS, and PD1 in a protein level (Fig. 1D), and *TOX2* expression showed a positive correlation with mRNA expression of T_FH_-associated genes including *PDCD1*, *BCL6*, *IL21*, *MAF*, *CXCR5*, and *CXCL13* (Fig. 1E). *PDCD1* (Pearson *R* = 0.975) and *BCL6* (Pearson *R* = 0.928) correlated highly with Tox2 mRNA levels (Fig. 1F). The Pearson *R* values for the correlation between *TOX2* and T_FH_-associated genes were more robust than those for the correlation between *TOX* or *BCL6* and T_FH_-associated genes (Fig. 1E). A strong correlation between *TOX2* and T_FH_ genes was also maintained in tonsillar CD4^+^ T cells even after TCR activation (fig. S1B), indicating that the correlation was independent of cell activity status.

Analysis with Western blot (WB) confirmed high expression of Tox2 protein by GC T_FH_ cells, but low by other tonsillar CD4^+^ T cell subsets (Fig. 1G). Tonsillar GC B cells, which highly express Bcl6, did not express Tox2 protein. In agreement with these observations, Tox2^+^ cells were largely limited to T cells within GCs by histology (Fig. 1H). Collectively, we confirmed that Tox2 is highly expressed by human tonsillar GC T_FH_ cells.

### Tox2 overexpression maintains the T_FH_ phenotype of GC T_FH_

**Fig. 2.**
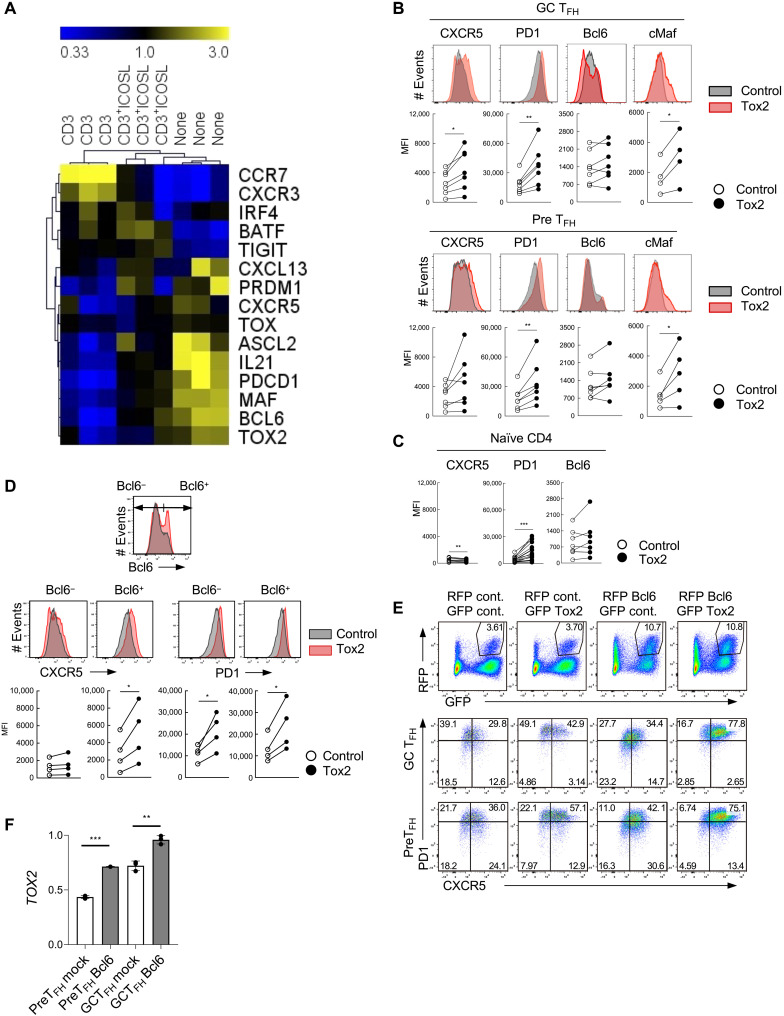
Tox2 overexpression maintains the T_FH_ phenotype of GC T_FH_. (**A**) mRNA expression of T_FH_-related genes in tonsillar GC T_FH_ cells after a 3-day stimulation with anti-CD3, anti-CD3^+^ICOSL, or none. Assessed by QuantiGene. Data are shown in a linear scale of the fold change from the median values. (**B**) Expression of T_FH_-related molecules by control and Tox2-overexpressing GC T_FH_ cells (top) and PreT_FH_ cells (bottom). Expression of CXCR5 and PD1 on the cell surface, and Bcl6 and cMaf in cell nucleus was analyzed. Representative flow data and the dataset of mean fluorescence intensity (MFI) of four to seven individual experiments are shown. (**C**) Expression of T_FH_-related molecules by control and Tox2 lentiviral overexpressed naïve CD4^+^ T cells. Dataset of MFI with six to seven individual experiments is shown. (**D**) Expression of CXCR5 and PD1 in Bcl6^+^ and Bcl6^−^ GC T_FH_ cells after transfection with a control vector or Tox2 expression vector. Bcl6^+^ and Bcl6^−^ cells were gated as shown in the top panel and examined for the expression of CXCR5 and PD1. Representative flow data (middle) and the dataset of MFI of four experiments (bottom) are shown. (**E**) Phenotype of GC T_FH_ and PreT_FH_ cells overexpressing Tox2 and/or Bcl6. GFP^+^RFP^+^ cells were analyzed for each culture condition. A representative result from two independent experiments. (**F**) *TOX2* mRNA expression form control and Bcl6 overexpressed tonsillar GC T_FH_ and PreT_FH_ cells. *N* = 3. *P < 0.05; **P < 0.01.

Ex vivo GC T_FH_ cells isolated from human tonsils were not phenotypically stable, as upon ex vivo stimulation with anti-CD3 mAb, GC T_FH_ cells lost the expression of many T_FH_-associated genes including *TOX2* within 3 days (Fig. 2A and fig. S2A). Although the ICOS signal pathway plays essential roles for T_FH_ cell differentiation and for their functions in GCs (*21*), stimulation of the ICOS signal pathway only partially inhibited the loss of T_FH_ genes in CD3-activated GC T_FH_ cells (Fig. 2A and fig. S2A). CD3-stimulated GC T_FH_ cells instead up-regulated the expression of CCR7 and CXCR3 (Fig. 2A), chemokine receptors promoting T cell egress from GCs. These observations show that ex vivo TCR stimulation induces human GC T_FH_ cells to spontaneously convert to non-T_FH_ cells, an observation consistent with the fact that GC T_FH_ cells are not terminally differentiated (*22*).

To determine the role of Tox2 in human T_FH_ cells, we generated a Tox2 expression vector [subcloned with green fluorescent protein (GFP) or red fluorescent protein (RFP) gene] (*23*) and transfected it into tonsillar GC T_FH_ and PreT_FH_ cells (fig. S2B). About 50% of the CD4^+^ T cells became positive for GFP or RFP (fig. S2C), and the expression of Tox2 protein was confirmed by WB (fig. S2D). We found that GC T_FH_ cells overexpressing Tox2 expressed higher levels of CXCR5, PD1, and cMaf than mock transfected (Fig. 2B). Unlike mouse CD4^+^ T cells (*24*), Tox2 overexpression did not increase the expression of Bcl6 in human tonsillar GC T_FH_, PreT_FH_, or naïve CD4^+^ T cells (Fig. 2, B and C). Tox2 overexpression did not induce the expression of *TOX* (fig. S2E). This function of Tox2 to induce T_FH_-related molecules was diminished when the functional domains, HMG box or interaction domain, were deleted (fig. S3, A and B). To further determine the function of Tox2 with Bcl6, we analyzed the promotion of CXCR5 and PD1 in cells positive and negative with the expression of Bcl6 within Tox2-overexpressing GC T_FH_ cells. We found that Tox2 overexpression promoted CXCR5 only in cells maintaining high Bcl6 (Fig. 2D). Furthermore, cotransfection of Tox2 and Bcl6 expression vectors resulted in the most robust generation of CXCR5^hi^PD1^hi^ cells from both GC T_FH_ and PreT_FH_ cells than the transfection of either vector (Fig. 2E). These observations suggest that cooperation of Tox2 and Bcl6 maintains the expression of CXCR5 and PD1 by GC T_FH_ cells in humans.

### T_FH_-promoting cytokines do not induce Tox2 in human CD4^+^ T cells

The strong correlation between *TOX2* and T_FH_-associated genes in tonsillar CD4^+^ T cells (Fig. 1D) and a functional cooperation of Tox2 and Bcl6 (Fig. 2, D and E) suggest that Tox2 and Bcl6 are simultaneously induced during T_FH_ cell differentiation. In line with this, a recent study in mice demonstrated that Bcl6 was sufficient to induce Tox2 and required for optimal Tox2 expression by murine CD4^+^ T cells (*24*). Murine naïve CD4^+^ T cells rapidly up-regulate *Tox2* in response to stimulation with cytokines IL-6 and IL-21 (*24*), which are signal transducer and activator of transcription 3 (STAT3)–activating cytokines that promote Bcl6-dependent T_FH_ cell differentiation (*6*). Bcl6 binds to the *Tox2* locus, and overexpression of Bcl6 induced Tox2 in mouse CD4^+^ T cells. Tox2 expression by Bcl6-deficient CD4^+^ T cells was diminished when cultured under T_FH_-promoting conditions (*24*).

In the experiments with human PreT_FH_ and GC T_FH_ cells, we found that Bcl6 overexpression increased *TOX2* but only slightly (Fig. 2F). When human cord blood naïve CD4^+^ T cells were stimulated in the presence of specific combinations of cytokines promoting T_FH_ cell differentiation in humans [IL-6, IL-23, and/or transforming growth factor–β (TGF-β)] (*25*), the stimulated cells up-regulated *BCL6* and *CXCR5* [both at mRNA and protein levels (*25*)], but they failed to up-regulate *TOX2* (fig. S4A). Cultured under different T_FH_-promoting conditions, anti–CD3+CD28 with IL-12 and/or sOX40L (*26*) did not induce human blood naïve and memory CD4^+^ T cells to increase *TOX2* (fig. S4B). STAT3-activating cytokines (IL-6 and IL-23) also failed to induce *TOX2* in human CD4^+^ T cells (fig. S4, A and B). Thus, unlike mouse CD4^+^ T cells, cytokine signals or Bcl6 is insufficient to induce *TOX2* by human CD4^+^ T cells.

### Tox2 overexpression inhibits spontaneous conversion of GC T_FH_ cells to non-T_FH_ cells

**Fig. 3.**
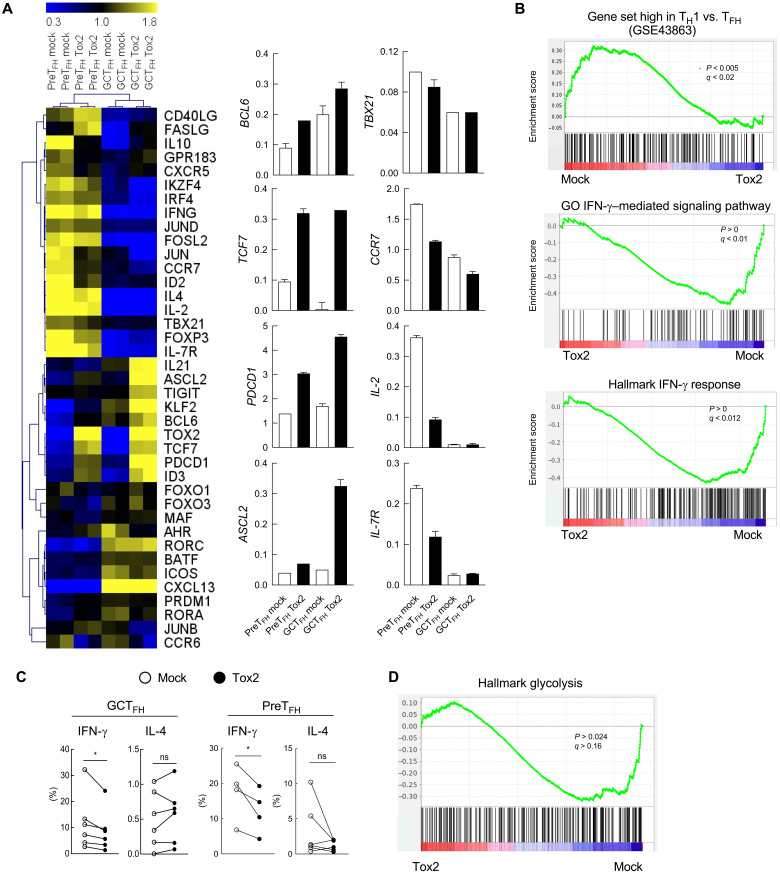
Tox2 inhibits GC T_FH_ cells to become T_H_1-like cells. (**A**) mRNA expression of T_FH_-related genes analyzed by QuantiGene in mock or Tox2-overexpressing GC T_FH_ cells and PreT_FH_ cells (left). CD4^+^CD19^−^RFP^+^ cells were sorted for mRNA analysis. The results of selected genes are shown on the right. *N* = 2. (**B**) Gene-set enrichment analysis (GSEA)in mock and Tox2-overexpressing tonsillar GC T_FH_ cells. Results with the gene set of up-regulated effector T_H_1 cells compared to effector T_FH_ cells (top), the gene set of down-regulated IFN-γ–mediated pathway (middle), and the gene set of IFN-γ response (bottom). (**C**) Percentage of cytokine-expressing cells by mock and Tox2-overexpressing GC T_FH_ and PreT_FH_ cells. Cells were stimulated with phorbol 12-myristate 13-acetate (PMA) + ionomycin for 6 hours, and the frequency of intracellular IFN-γ^+^ and IL-4^+^ cells was analyzed. Dataset of four to seven experiments is shown. (**D**) GSEA analysis in mock and Tox2-overexpressing tonsillar GC T_FH_ cells with the gene set of down-regulated glycolysis pathways.

To further gain insights into the role of Tox2 in human GC T_FH_ cells, we analyzed the global transcriptional profiles of Tox2-overexpressing GC T_FH_ cells. Tox2 overexpression in GC T_FH_ cells resulted in higher expression of T_FH_-associated genes than in mock-transfected cells, including *IL21*, *ASCL2*, *TIGIT*, *KLF2*, *BCL6*, *TCF7*, *PDCD1*, and *ID3* (Fig. 3A). The high expression of T_FH_-associated genes by Tox2 overexpression was more evident in GC T_FH_ cells than in PreT_FH_ cells. By contrast, mock-transfected GC T_FH_ cells expressed more pronounced T_H_1-associated genes and IFN-γ–responsive genes (Fig. 3B). Consistently, mock-transfected GC T_FH_ and PreT_FH_ cells expressed more IFN-γ, but not IL-4, upon polyclonal stimulation than Tox2-transfected cells (Fig. 3C). These results indicate that Tox2 insulates TCR-stimulated GC T_FH_ cells from becoming T_H_1-like cells.

In mice, Tox2 was shown to inhibit T-bet by directly binding its gene regulatory elements (*24*). In contrast, a previous study with human natural killer cells showed that Tox2 overexpression increases T-bet expression (*27*), and therefore, the effect of Tox2 on T-bet in human cells remains unclear. In human tonsillar CD4^+^ T cells, Tox2 overexpression did not affect T-bet expression (encoded by *TBX21*) (Fig. 3A). This suggests that the inhibition of spontaneous conversion into T_H_1-like cells by Tox2 overexpression was not due to increased expression level of T-bet. A recent study showed that inhibiting glycolysis by treatment with 2-deoxy-d-glucose (2DG) induces human T cells to up-regulate Tox2 (*28*). Because 2DG treatment also induces Bcl6 (*29*), which inhibits the glycolysis program in T cells (*30*), inhibition of glycolysis might play an important role for the maintenance of GC T_FH_ cells. This is in line with the observation that T_FH_ cells are less reliant on glucose than T_H_1 cells (*31*). We found that mock transfection of GC T_FH_ cells increased expression of glycolysis genes compared to Tox2-transfected cells (Fig. 3D). Thus, it is possible that Tox2 might protect GC T_FH_ cells from spontaneous T_H_1 conversion by maintaining a low glycolysis profile.

### Tox2-deficient mice display impaired secondary T_FH_ cell response

As observed in humans, GC T_FH_ cells highly express Tox2 in mice, and the expression correlates with the expression of T_FH_-related molecules (fig. S5A) (*24*). To determine the biological significance of Tox2 in T_FH_ cell response in vivo, we generated Tox2-deficient mice by CRISPR by deleting the sequence from exon 4 to exon 8 of the *Tox2* gene, which covers the functional HMG box domain and a part of the interaction domain (fig. S5B). The expression of Tox2 in deficient mice was confirmed at a protein level (fig. S5C). Unlike Tox-deficient mice that lack CD4^+^ T cells (*32*), Tox2 deficiency did not affect the development and maturation of CD4^+^ and CD8^+^ T cell populations in the thymus and the spleen in homozygous mice (fig. S5D), a consistent observation with a recent report (*24*).

**Fig. 4.**
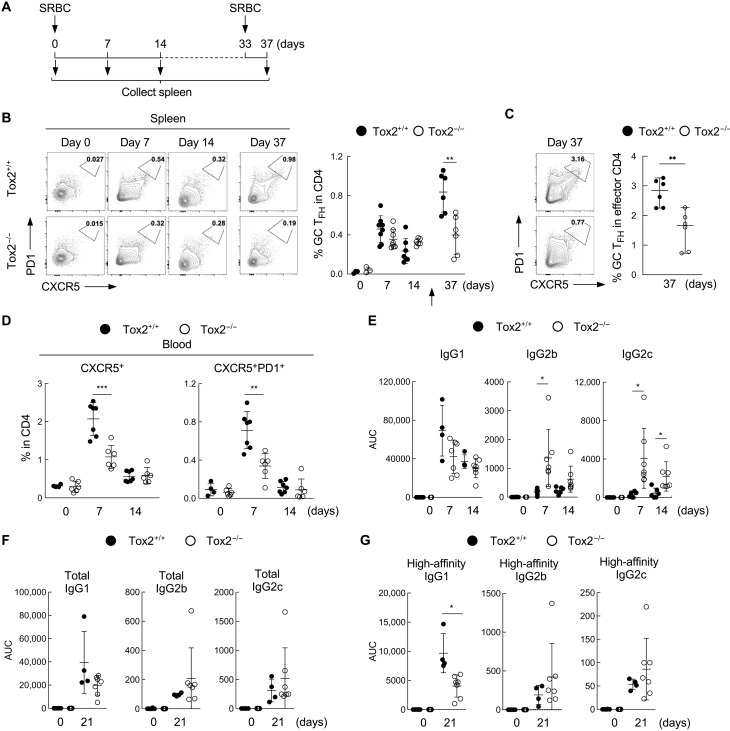
Tox2-deficient mice failed to induce secondary T_FH_ cell response. (**A**) Experimental design of mice immunization. C57BL/6 Tox2^+/+^ and Tox2^−/−^ mice were intraperitoneally immunized with 1 × 10^9^ SRBCs. Blood cells were collected before immunization and at days 7 and 14 after immunization. At 33 days after primary immunization, mice were reimmunized with 5 × 10^8^ SRBC. Spleen cells were collected 4 days after second immunization. (**B**) Frequency of PD1^hi^CXCR5^hi^ GC T_FH_ cells in spleen CD4^+^ T cells at each indicated time point. The arrow indicates the time point for secondary immunization. Representative flow data (left) and the dataset of three to eight mice are shown (right). (**C**) Frequency of PD1^hi^CXCR5^hi^ GC T_FH_ cells within CD62L^lo^CD44^hi^ effector CD4^+^ T cells at day 37. Representative flow data (left) and the dataset of six mice are shown (right). (**D**) Frequency of CXCR5^+^ and CXCR5^+^PD1^+^ CD4^+^T cells in the blood at indicated time points. *N* = 6. (**E**) Total anti-NP IgG1, IgG2b, and IgG2c measured with NP14-BSA in the serum at indicated time points before and after NP-SRBC immunization. Each symbol represents the result from an individual mouse. Three to eight WT and knockout mice were used in each experiment. (**F**) Total anti-NP IgG1, IgG2b, and IgG2c in the serum of NP-SRBC immunized mice at day 21 after immunization. *N* = 3 to 8. (**G**) High-affinity anti-NP IgG1, IgG2b, and IgG2c measured with NP2-BSA in the serum of NP-SRBC immunized mice at day 21 after immunization. *N* = 3 to 8. *P < 0.05; **P < 0.01.

To examine whether Tox2 deficiency affects T_FH_ cell response in vivo, we immunized wild-type (WT) and Tox2-deficient mice with sheep red blood cells (SRBCs) conjugated with the hapten 4-hydroxy-3-nitrophenylacetyl (NP) intraperitoneally and analyzed GC T_FH_ and GC B cell populations in the spleens (Fig. 4A). The frequency of CXCR5^hi^PD1^hi^ GC T_FH_ cells in the spleens did not differ between WT and Tox2-deficient mice at day 7 or day 14 after immunization (Fig. 4B). However, 4 days after secondary immunization with SRBC (day 37 after immunization), Tox2-deficient mice failed to increase GC T_FH_ cells (Fig. 4, B and C), suggesting an impairment in the induction of the secondary T_FH_ cell response.

Previous studies showed that a fraction of GC T_FH_ cells and T_FH_ precursors induced by immunization egress from the draining lymph nodes (LNs) and become circulating memory T_FH_ cells (*11*, *17*, *33*, *34*). We hypothesized that impaired secondary T_FH_ cell response might be associated with an impaired generation of cT_FH_ cells. Levels of CXCR5^+^ and CXCR5^+^PD1^hi^ cT_FH_ cells were significantly diminished in Tox2-deficient mice at day 7 after immunization (Fig. 4D).

### Tox2-deficient mice failed to induce durable high-affinity Ab response

To determine the biology of Tox2 deficiency in GC responses, we measured the serum levels of NP hapten–specific IgG1, IgG2b, and IgG2c. Tox2-deficient mice developed larger amounts of NP-specific IgG2b and IgG2c at days 7 and 14 after immunization than WT mice (Fig. 4E). WT mice generated relatively higher NP-specific IgG1 (Fig. 4, E and F), specifically at day 21 after immunization (Fig. 4G). By contrast, the generation of high-affinity IgG2b and IgG2c Abs was similar between WT and Tox2-deficient mice (Fig. 4G). Thus, Tox2-deficient mice could generate larger amounts of IgG2b and IgG2c than WT mice at day 7 after immunization, but this did not result in larger amounts of high-affinity IgG2b and IgG2c at day 21 after immunization.

### T_FH_ cell phenotype was impaired in Tox2-deficient mice infected with an influenza virus

**Fig. 5.**
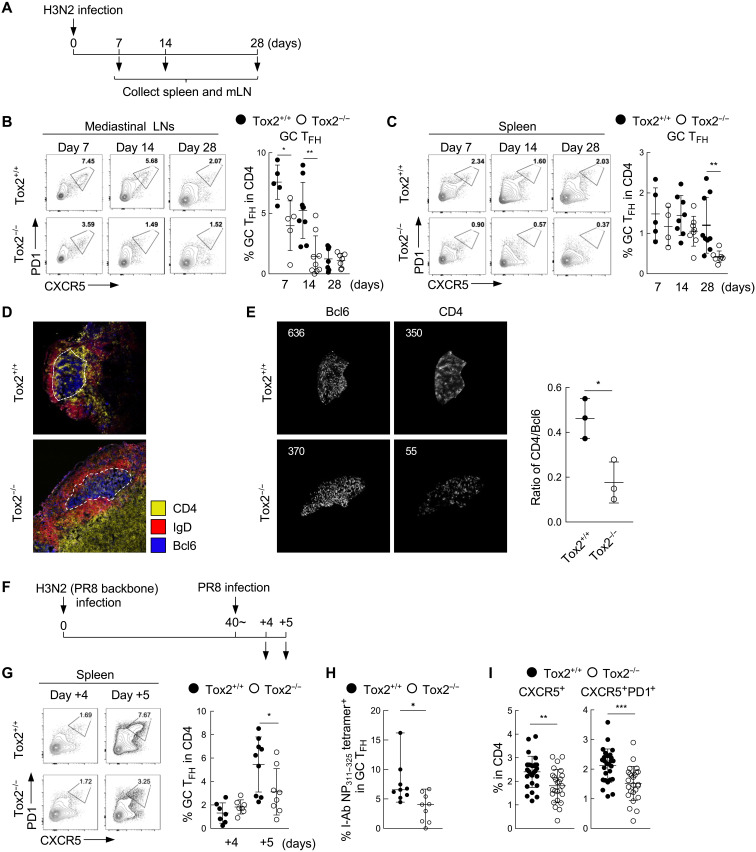
T_FH_ cell response is impaired in Tox2-deficient mice upon infection with influenza virus. (**A**) Experimental protocol of influenza virus infection. C57BL/6 Tox2^+/+^ and Tox2^−/−^ mice were intranasally infected with H3N2 ×31 (PR8 backbone: HA and NA from A/Aichi/2/68). Lung mLNs and spleen cells were collected at 0, 14, and 28 days after infection to analyze CD4^+^ T cell populations by FACS. (**B** and **C**) Frequency of PD1^hi^CXCR5^hi^ GC T_FH_ cells in mLNs (B) and spleen (C) at the indicated time points after H3N2 ×31 infection. Representative flow data (left) and the dataset of four to nine mice are shown (right). (**D**) Immunofluorescence images of the frozen mLN sections from Tox2^+/+^ and Tox2^−/−^ mice 14 days after H3N2 ×31 infection. Bcl6 in blue, IgD in red, and CD4 in yellow. (**E**) Bcl6^+^ and CD4^+^ cells within GC are shown in the left. The ratio of Bcl6^+^ cells/CD4^+^ cells in GC is shown in the right panel. Analyzed by ImageJ. (**F**) Experimental protocol for heterosubtypic virus challenge. Tox2^+/+^ and Tox2^−/−^ mice were intranasally infected with H3N2 ×31 first and reinfected by H1N1 PR8. Spleen cells were collected 4 and 5 days after secondary infection to analyze CD4^+^ T cell populations by FACS. (**G**) Frequency of PD1^hi^CXCR5^hi^ GC T_FH_ cells within Foxp3-CD4^+^ T cells in the spleen after H1N1 PR8 reinfection. Representative flow data (left) and the dataset of four to nine mice are shown (right). (**H**) Frequency of I-Ab Influenza NP_311–325_ tetramer-positive CD4^+^ T cells within PD1^hi^CXCR5^hi^ GC T_FH_ cells in the spleen after H1N1PR8 reinfection. *N* = 9. (**I**) CXCR5^+^ (left) and CXCR5^+^PD1^+^ (right) CD4^+^ cells in the blood in Tox2^+/+^ and Tox2^−/−^ mice at day 7 after primary H3N2 influenza virus infection. *N* = 25 to 27. *P < 0.05; **P < 0.01; ***P < 0.001.

Ab production and T_FH_ responses are fundamental for protection from virus infection. Next, we examined whether Tox2-deficient mice can mount intact T_FH_ cell response upon infection with an influenza virus. Mice were intranasally infected with an X-31 H3N2 influenza virus [A/Puerto Rico/8/34, PR8 backbone with hemagglutinin (HA) and neuraminidase (NA) derived from A/Aichi/2/68] and analyzed for the generation of T_FH_ cells in mediastinal LNs (mLNs) and the spleens (Fig. 5A). There were no differences in body weight after infection (fig. S6A). The frequencies of GC T_FH_ cells in the mLNs were significantly lower at days 7 and 14 after infection in Tox2-deficient mice than in WT mice (Fig. 5B). However, in the spleens, no difference was observed in the frequency of GC T_FH_ cells between WT and Tox2-deficient mice at day 7 after infection. The population of GC T_FH_ cells decreased more rapidly in Tox2-deficient mice and became significantly lower at day 28 after infection as compared to WT mice (Fig. 5C). The frequency of FoxP3^+^ T follicular regulatory (T_FR_) cells was similar between Tox2-deficient mice and WT mice in both mLNs and spleens (fig. S5, B and C), suggesting that the low frequency of GC T_FH_ cells in Tox2-deficient mice in mLNs and spleens was unlikely due to an increased T_FR_ response.

Unexpectedly, the frequency of GC B cells did not differ between Tox2-deficient mice and WT mice in the mLNs or spleen at day 14 or 28 after infection (fig. S5, D and E). Immunofluorescent microscopy analysis confirmed the formation of GCs in the mLNs of both Tox2-deficient and WT mice (Fig. 5D). However, when we analyzed the frequency of T_FH_ cells located inside GCs by examining the number of CD4^+^ T cells within Bcl6^+^ GCs, we found that Tox2-deficient mice displayed significantly fewer T_FH_ cells per constant Bcl6^+^ GC area (Fig. 5E), indicating the generation of less GC T_FH_ cells in Tox2-deficient mice. Nonetheless, unlike an immunization with SRBC, there were no substantial differences in the titers of H3 HA-specific IgG isotypes between Tox2-deficient and WT mice (fig. S5, F and G). Thus, impaired T_FH_ cell phenotype in influenza-infected Tox2-deficient mice did not largely affect Ab responses following influenza virus infection, probably because the primary mechanism for Ab production in influenza virus infection is via an extrafollicular pathway (*35*).

To examine whether the secondary T_FH_ cell responses are also altered in Tox2-deficient mice, we challenged the mice by infection with the X-31 strain and then reinfected the mice 40 days later with an H1N1 influenza virus strain (A/Puerto Rico/8/34, PR8) (Fig. 5F). The X-31 and PR8 influenza virus strains share the same genetic backbone but encode different HA and NAs, which are the primary targets of neutralizing Ab responses. Consistent with the immune responses following SRBC immunization, induction of secondary response of influenza virus by PR8 infection decreased GC T_FH_ cells in the spleens at day 5 after infection of Tox2-deficient mice as compared to WT mice (Fig. 5G). To confirm a recall of T_FH_ cell response, we analyzed the frequency of I-Ab influenza NP_311–325_ tetramer^+^ cells within GC T_FH_ cells. The sequence of NP_311–325_ is shared between X-31 H3N2 influenza virus and PR8. We found that the frequency of tetramer-positive T_FH_ cells was significantly lower in Tox2-deficient mice (Fig. 5H). As observed for the SRBC immunization model, Tox2-deficient mice displayed less cT_FH_ cells in the blood at day 7 after primary infection with H3N2 (Fig. 5I). Collectively, both primary and secondary T_FH_ responses after influenza infection were diminished in Tox2-deficient mice.

**Fig. 6.**
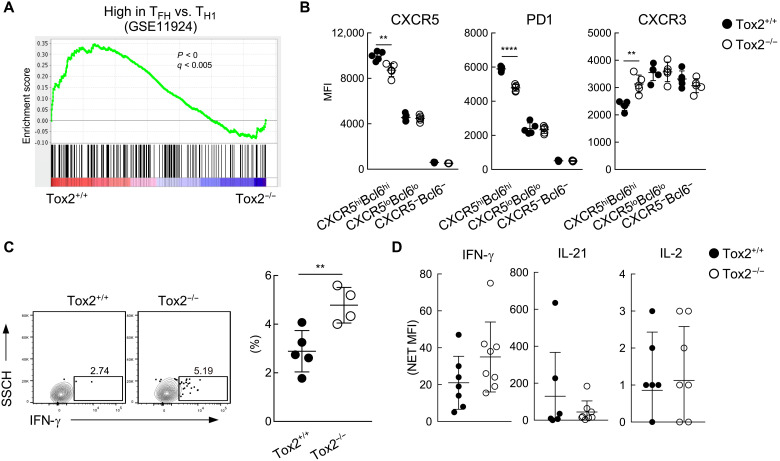
GC T_FH_ cells in Tox2-deficient mice exhibited less T_FH_ gene signature. (**A**) GSEA analysis in Tox2^+/+^ and Tox2^−/−^ PD1^hi^CXCR5^hi^ GC T_FH_ cells with the gene set of up-regulated T_FH_ compared to effector T_H_1. (**B**) MFI of CXCR5, PD1, and CXCR3 in CXCR5^hi^Bcl6^hi^, CXCR5^lo^Bcl6^lo^, and CXCR5^−^Bcl6^−^ CD4^+^ T cells. *N* = 4 to 5. (**C**) IFN-γ expression of spleen CD4^+^ T cells 7 days after H3N2 infection. Splenocytes were incubated with heat-inactivated H3N2 influenza virus for 12 hours followed by another 12-hour culture in the presence of GolgiPlug and brefeldin. IFN-γ expression by splenic CD4^+^ T cells was assessed by FACS. Representative flow data (left panel) and the dataset of four to five mice are shown (right). (**D**) IFN-γ, IL-21, and IL-2 amount in serum from mice 7 days after SRBC immunization. **P < 0.01; ****P < 0.001.

To further analyze the function of Tox2 in Tox2-deficient mice, we collected CXCR5^hi^PD1^hi^ T_FH_ cells from both WT and Tox2-deficient mice for DNA microarray analysis. Transcript analysis revealed a reduced T_FH_ gene signature for GC T_FH_ cells from Tox2-deficient mice as compared to GC T_FH_ cells from WT mice (Fig. 6A). By flow cytometry analysis, CXCR5^hi^Bcl6^hi^ T_FH_ cells in Tox2-deficient mice also expressed lower levels of CXCR5 and PD1, and higher levels of CXCR3, a T_H_1-type chemokine receptor, as compared to WT mice (Fig. 6B). Furthermore, the frequency of influenza virus–specific IFN-γ^+^ CD4^+^ T cells was significantly higher in the spleen of Tox2-deficient mice than that of WT mice (Fig. 6C). Serum levels of IFN-γ were relatively higher at day 7 after infection in Tox2-deficient mice than in WT mice (Fig. 6D). These observations confirm that the maturation of GC T_FH_ cells was impaired in Tox2-deficient mice, whereas the T_H_1 cell response was increased.

### Diminished T_FH_ cell phenotype by Tox2 deficiency is CD4^+^ T cell intrinsic

**Fig. 7.**
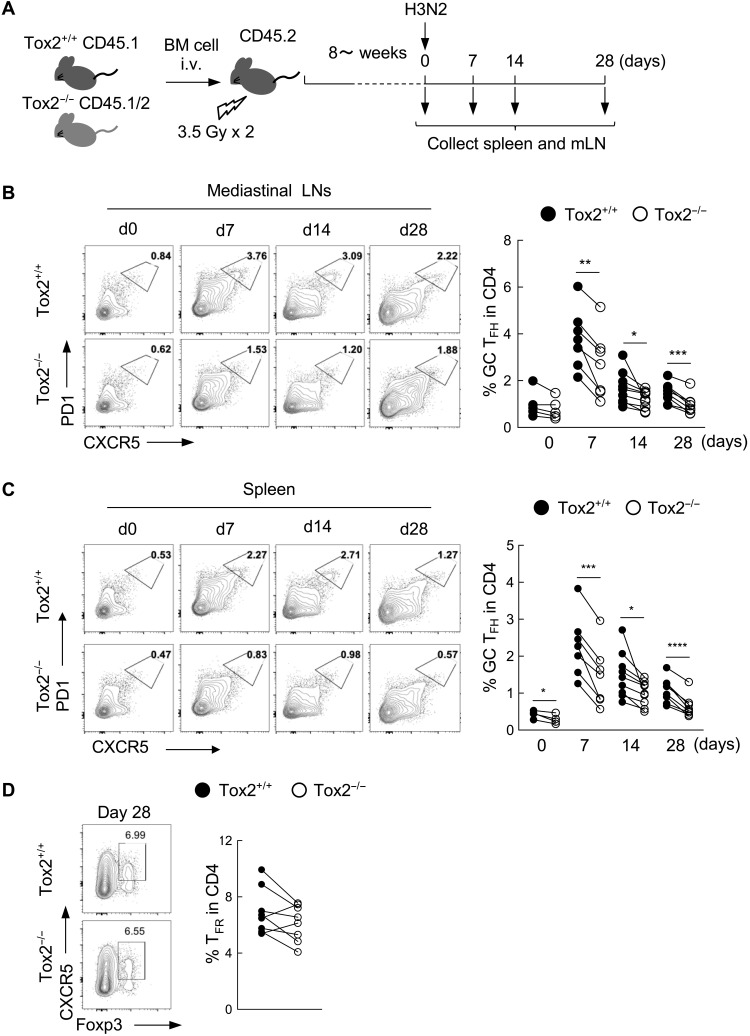
Diminished T_FH_ cell phenotype by Tox2 deficiency is CD4^+^ T cell intrinsic. (**A**) Experimental protocol of the generation of mix BM chimera and H3N2 infection. The BM from C57BL/6 Tox2^+/+^ and Tox2^−/−^ mice with the indicated congenic marker was transferred to irradiated C57BL/6 Tox2^+/+^ mice intravenously. After more than 8 weeks, mice were intranasally infected with H3N2. mLNs and spleen cells were collected at days 0, 7, 14, and 28 after infection to analyze cell populations by FACS. (**B** and **C**) Frequency of PD1^hi^CXCR5^hi^ GC T_FH_ cells in mLN (B) and spleen (C) at indicated time points. Representative flow data (left) and the dataset of 6 to 12 mice are shown (right). (**D**) Frequency of CXCR5^+^Foxp3^+^ T_FR_ cells in spleen at indicated time points. Representative flow data (left) and the dataset of seven mice are shown (right).

Analysis of publicly available RNA sequencing data suggested that expression of Tox2 is reported to be expressed by other immune cells including innate lymphoid cells (ILCs) and macrophages in mice (www.immgen.org). To examine whether the impaired T_FH_ cell generation by Tox2-deficient mice was CD4^+^ T cell intrinsic, we generated bone marrow (BM) chimeric mice by transferring BM cells from Tox2-deficient mice (CD45.1/2) and WT mice (CD45.1) into irradiated mice (CD45.2). BM chimeric mice were then infected with the X-31 H3N2 influenza virus and examined for the phenotype of GC T_FH_ cells in mLNs and spleens (Fig. 7A). The frequency of WT (CD45.1) and Tox2-deficient (CD45.1/2) CD4^+^ T cells was the same between different time points after virus infection (fig. S7). The frequency of GC T_FH_ cells in Tox2-deficient CD4^+^ T cells was significantly lower in both mLNs and spleens at days 7, 14, and 28 after infection (Fig. 7, B and C). There was no difference in the frequency of T_FR_ cells in the spleens derived from Tox2-deficient CD4^+^ T cells and WT CD4^+^ T cells (Fig. 7D). These results indicate that Tox2 is intrinsically required for maturation of GC T_FH_ cells.

## DISCUSSION

In this study, we analyzed the role of Tox2 in T_FH_ cell responses using ex vivo human tonsillar GC T_FH_ cells and in vivo mouse models. Our study showed that Tox2 is required to maintain GC T_FH_ cells and generate memory T_FH_ cells, while several species-specific features exist.

Recent evidence in mice showed that Tox2 and Tox display redundant roles for T_FH_ cell differentiation (*24*) and CD8^+^ T cell exhaustion (*36*). In human T_FH_ cell biology, Tox2 appears to play a dominant role than Tox. Whereas Tox expression was high in human tonsillar GC T_FH_ cells (fig. S1C), Tox2 expression was more restricted to GC T_FH_ cells among human blood and tonsillar CD4^+^ T cells (fig. S1). Furthermore, the overall correlation of *TOX2* with T_FH_-associated genes was stronger than that of *TOX*. Among human blood and tonsillar CD4^+^ T cells, high Tox2 expression was restricted to tonsillar GC T_FH_ cells. Whereas tonsillar PreT_FH_ cells expressed a higher level of Tox2 than any blood CD4^+^ T cell subsets analyzed, Tox2 expression in PreT_FH_ cells was substantially lower than that in GC T_FH_ cells in tonsils at both transcriptional and protein levels. *TOX2* expression in tonsillar CD4^+^ T cell subsets positively correlated with many T_FH_-associated genes, including *PDCD1*, *MCL6*, *IL21*, *MAFB*, *MAF*, *CXCR5*, *TOX, CXCL13*, and *ICOS*, but negatively correlated with non-T_FH_ genes, including *TBX21*, *FOXP3*, and *ID2*. These observations suggest that Tox2 is highly integrated within the differentiation and maturation programs of human T_FH_ cells.

The observation that TCR stimulation reduces Tox2 expression of GC T_FH_ cells ex vivo does not contradict the fact that GC T_FH_ cells maintain high Tox2 expression. Probably, such difference could be explained by the difference in TCR signal strength. Whereas ex vivo TCR stimulation with anti-CD3 is strong (potentially even at an unphysiological level), GC T_FH_ cells receive only transient and short TCR signals upon encounter with GC B cells (*37*).

By using a Tox2 overexpression system, we demonstrated that Tox2 promoted tonsillar GC T_FH_ cells to maintain T_FH_ markers including CXCR5, PD1, and cMaf. Whereas ex vivo activated tonsillar GC T_FH_ cells rapidly lose T_FH_ molecules and the gene signature, Tox2 overexpression maintained the expression of T_FH_ genes, including *IL21*, *ASCL2*, *TIGIT*, *KLF2*, *BCL6*, *TCF7*, *PDCD1*, and *ID3*. Thus, similar to Bcl6 (*38*), these data support our findings that Tox2 contributes to T_FH_ cell maturation and/or maintenance of human T cells. Furthermore, Tox2 and Bcl6 synergized in the expression of CXCR5 and PD1, suggesting that Tox2 cooperates with Bcl6 during the maturation of human T_FH_ cells. Bcl6 is the T_FH_ lineage–defining transcriptional factor that represses other transcriptional factors fundamental for the differentiation of other T_H_ linage cells, such as T-bet in T_H_1 cells (*6*). Tox2 overexpression prevented spontaneous conversion of ex vivo human GC T_FH_ cells into T_H_1-like cells, suggesting that Tox2 shares its functions with Bcl6. Unlike Bcl6, Tox2 inhibits T_H_1 program independent of T-bet in humans. Although further studies are required for the precise mechanism, our study suggests that Tox2-overexpressed human T_FH_ cells expressed less glycolysis genes, a feature associated with the maintenance of mature GC T_FH_ cells.

In mice, Tox2 seems to be involved in the early stage of T_FH_ cell differentiation, as STAT3 and Bcl6 cooperate to drive Tox2 expression during the initial T_FH_ cell differentiation (*24*). The significance of this pathway is unclear in humans because, although Bcl6 overexpression slightly increased Tox2 expression in tonsillar T_FH_ cells, neither STAT signals nor Bcl6 was sufficient to induce strong expression of Tox2 in humans. Stimulation of CD4^+^ T cells under T_FH_-promoting cytokine conditions, including IL-12 and IL-23, which activate STAT3 and STAT4, failed to induce Tox2 expression despite Bcl6 expression. Given that inhibiting glycolysis causes human T cells to up-regulate Tox2 (*28*), human T cells may be more dependent than mouse CD4^+^ T cells for Tox2 expression on the factors derived from GC microenvironment where the resource of glucose is limited (*39*, *40*).

Despite some differences in the expression and the role of Tox2 between humans and mice, to gain insights into the physiological function of Tox2 in animals in vivo, we generated Tox2-deficient mice and induced T_FH_ cell response in SRBC immunization and against influenza virus infection. A substantial reduction of T_FH_ cells was observed after the secondary challenge in both SRBC and influenza virus infection. Because the T_FH_ cells in the secondary immune responses are originated mostly from memory T_FH_ cells (*18*), an impaired secondary T_FH_ cell response in Tox2-deficient mice was likely due to impaired generation of memory T_FH_ cells. Consistently, we found a significant decrease in blood cT_FH_ cells, which contain a precursor of memory T_FH_ cells. Last, by using a BM chimera approach, we showed that impaired phenotype of T_FH_ cells in Tox2-deficient mice in primary influenza virus infection was due to a CD4^+^ T cell–intrinsic effect of Tox2. Thus, our observations in the experiment with Tox2-deficient mice are similar with a recent report (*24*) and further extend our knowledge on the role of Tox2 into the generation of T_FH_ cell memory.

In conclusion, our study highlights the role of Tox2 for the maintenance of GC T_FH_ cell biology and the generation of T_FH_ cell memory. The precise mechanism by which Tox2 expression is induced during human T_FH_ cell differentiation remains to be established. Because durable T_FH_ cell response is required for high-affinity efficient Ab response, determination of the pathways and molecular mechanisms for Tox2 induction in human T_FH_ cells will provide novel insights into designs for novel vaccines.

## MATERIALS AND METHODS

### Human tonsillar and blood T and B cells

Tonsillar CD4^+^ T cells and B cell subsets were isolated as described previously (*41*). When indicated, T and B cell populations were cultured in RPMI 1640 medium (Gibco) supplemented with penicillin G/streptomycin (1%, Gibco), gentamicin (50 μg/ml; Gibco), timentin (125 μg/m; PlantMedia), Hepes solution (25 mM; Gibco), sodium pyruvate (1 mM; Gibco), nonessential amino acid (1%; Gibco), 50 μM β-mercaptoethanol (Sigma-Aldrich), and 10% heat-inactivated fetal calf serum (FCS) in the presence of endotoxin-reduced staphylococcal enterotoxin B (SEB) (1 μg/ml; Sigma-Aldrich) in U-bottomed 96-well plates. For CD3 and ICOSL stimulation, tonsillar CD4^+^ T cells were cultured with CD32-transfected L cells or CD32 and ICOSL cotransfected L cells in the presence of anti-CD3 for 72 hours (*42*). After 72 hours, CD4^+^CD3^+^ cells were sorted by fluorescence-activated cell sorting (FACS) for transcriptional profiling. The transcript of the isolated cells was analyzed by microarray (Illumina) or NanoString (*25*). All experiments were approved by and performed according to the guidelines of the Icahn School of Medicine at Mount Sinai Institutional Review Boards (IRB-17- 01710).

### HDV production and transfection

Human Tox2 sequence was subcloned into HIV-derived vector (HDV)–expressing vector as Tox2 overexpression HDV vector. Lentivirus was produced by transfecting 293T cells with HDV vector and vesicular stomatitis virus G plasmids. The virus-containing supernatants were collected at 48 and 72 hours after transfection and passed through a 0.45-mm filter. The virus preparation was then concentrated with an Amicon Ultra 100-kDa filter and stored at −80°C until use. For the transfection, cells were plated in flat-bottomed 96-well and transduced at 5 to 10 multiplicities of infection. The transfection efficiency was increased by spinning at 1200*g* for 2 hours at room temperature. For T-B cocultured cells, cells were cultured in the presence of SEB (1 μg/ml) 2 days before transfection. Cells were plated in a 96-well U-bottom plate after transfection and cultured for 7 days for further analysis. GFP- or RFP-expressing HDV vector was obtained from D. Unutmaz (*43*).

### Western blotting

For human cells, total proteins were extracted from sorted tonsil CD4^+^ T cell populations. For mice cells, total proteins were extracted from freshly isolated splenocyte or effector CD4^+^ T cells. Radioimmunoprecipitation assay buffer (Sigma-Aldrich) supplemented with 1% protease inhibitor mixture (Sigma-Aldrich) was used to extract proteins. Equal amounts of protein per sample were separated on NuPAGE (Invitrogen) 4 to 12% bis-tris gradient gels and transferred to polyvinylidene difluoride (PVDF) membranes (Invitrogen). Membranes were incubated with anti-Tox2 (LS-C29895, LSBio) for human, anti-Tox2 (21162AP, ProteinTech) for mice, and anti-Bcl6 (K112-91, BD Biosciences) followed by horseradish peroxidase (HRP)–conjugated anti-mouse or anti-rabbit (Santa Cruz Biotechnology). Equal protein loading was confirmed using anti–glyceraldehyde-3-phosphate dehydrogenase (GAPDH) (GAPDH-71.1, Sigma-Aldrich).

### Flow cytometry

Human helper T cells were stained with anti-CD4 (RPAT4, BioLegend), anti-CD3 (HIT3A, BioLegend), anti-PD1 (EH12-27, BioLegend), anti-CXCR5 (RF8B2, BD Biosciences), and anti-ICOS (ISA-3, eBioscience). For intranuclear staining, cells were fixed for 30 min at 4°C with True-Nuclear buffer (BioLegend) and then incubated for 30 min at 4°C with anti-Bcl6 (K112-91, BD Biosciences) and anti-cMaf (sym0F1, Invitrogen) in Perm/Wash buffer (BioLegend). Mouse single-cell suspended mLNs or spleens were stained with anti-CD3 (145-2C11, BioLegend), anti-CD4 (GK1.5, BioLegend), anti-CD8 (53-6.7, BioLegend), anti-CD19 (6D5, BioLegend), anti-CXCR5 (L138D7, BioLegend), anti-PD1 (RMP1-30, BioLegend), and anti-CXCR3 (CXCR3-173, BD Biosciences). For intranuclear staining, cells were fixed for 30 min at 4°C with True-Nuclear buffer (BioLegend) and then incubated for 30 min at 4°C with anti-Bcl6 (K112-91, BD Biosciences) and anti-Foxp3 (150D, BioLegend) in Perm/Wash buffer (BioLegend). All samples were incubated with LIVE/DEAD Fixable Aqua or LIVE/DEAD Fixable Blue (Invitrogen) to exclude dead cells from the analysis. Cells were acquired on BD FACSCanto II or BD LSR II (BD Biosciences). Expression of each molecules was assessed with FlowJo software (TreeStar) in CD4^+^ T cell subset or for cultured cells among activated (FSC^hi^SSC^hi^) helper T cells. To detect the expression of Tox2 in human tonsils, PrimeFlow RNA assay (Invitrogen) was performed following the manufacturer’s instructions.

### Immunohistochemistry

For human tonsils, frozen sections of tonsils, 6 μm in thickness, were fixed with cold acetone and stained with anti-CD3 (UCHT1l, BD Biosciences) and anti-Tox2 (LS-C29895, LSBio) followed by Alexa Fluor 568–conjugated anti-mouse IgG1 (A-21124, Molecular Probes) and Alexa Fluor 488–conjugated anti-rabbit (A-11070, Molecular Probes). Last, sections were counterstained for 2 min with 3 μM DAPI (4′,6-diamidino-2-phenylindole). For mice spleen, frozen sections, 6 μm in thickness, were fixed with True-Nuclear buffer (BioLegend) and stained with anti-CD4 (GK 1.5) and anti-Bcl6 (K112-91, BD Biosciences) at 4°C overnight followed by 20 min at room temperature for anti-IgD (11-26c.2a, BioLegend) in Perm/Wash buffer (BioLegend). Sections were mounted with ProLong Glass Antifade Mountant with NucBlue (Invitrogen) for DAPI counterstaining and sealing. Slide images were observed under a Leica SP5 confocal microscope with a PlanApo 20× objective (numerical aperture, 0.7) and a PlanApo 40× objective (numerical aperture, 1.25).

### Mice

Tox2^−/−^ mouse strain was generated by Taconic by deleting exons 4 to 8, which contain the HMG box and interaction domain by CRISPR with C57BL/6 background. Two single-guide RNAs (sgRNAs) were selected from candidate sgRNAs by their position and a low number of predicted off-targets. The off-target analysis is based on the GRCm34/mm10 assembly. After administration of hormones, superovulated C57BL/6NTac females were mated with C5BL/6NTac males. One-cell-stage fertilized embryos were isolated from the oviducts at days post coitum (dpc) 0.5. For microinjection, the one-cell-stage embryos were placed in a drop of M2 medium under mineral oil. A microinjection pipette with an approximate internal diameter of 0.5 μm (at tip) was used to inject the mixed nucleotide preparation into the pronucleus of each embryo. After recovery, 25 to 35 injected one-cell-stage embryos were transferred to one of the oviducts of 0.5 dpc, pseudo-pregnant NMRI females. Proximal and distal CRISPR RNA/transactivating CRISPR RNA hybrids, instead of sgRNA molecules, were coinjected into C57BL/6NTac zygotes along with Cas9 protein. After the first generation was born, genomic DNA was extracted from tail biopsies and analyzed by polymerase chain reaction (PCR). The PCR amplicons were analyzed by using a Caliper LabChip GX device for genotyping. All mice were female and age-matched and were between 8 and 12 weeks old. Mice were bred and maintained under specific pathogen–free conditions in the animal facility at Icahn school of Medicine at Mount Sinai. All experiments were performed in compliance with the Institutional Animal Care and Use Committee (IACUC) guidelines.

### Mice SRBC immunization and influenza virus infection

For SRBC immunization, SRBCs were obtained from Innovative Research and washed with phosphate-buffered saline (PBS) until the supernatant was clear. Mice were immunized intraperitoneally with 1 × 10^9^ SRBC to induce T-dependent GC response. For influenza virus infection, mice were challenged intranasally with 50 μl of either a sublethal dose [20 plaque-forming units (PFU)] of influenza virus AH3N2 ×31 (PR8 backbone: HA and NA from A/Aichi/2/68) (*44*) or 100 PFU of influenza virus AH1N1 (A/Puerto Rico/8/34, PR8) at egg-grown preparation diluted in PBS under mild ketamine/xylazine anesthesia. Body weight loss was measured on a daily basis as a readout for morbidity. All experiments were approved and performed according to the guidelines of the Icahn School of Medicine at Mount Sinai IACUC (IACUC-2016-0074). The methods used were carried out in accordance with the approved guidelines. Mice were euthanized when they had reached the ethical end point of 20% body weight loss.

### Mix BM chimera

C57BL/6 Tox2^+/+^ and Tox2^−/−^ mice with congenic transfer marker BM cells (1 × 10^6^ cells each) were transferred to C57BL/6 Tox2^+/+^ mice irradiated with 3.5 Gy two times with 3-hour interval intravenously. After more than 8 months, mice were infected intranasally with H3N2. Lung draining LN and spleen cells were collected at days 0, 14, and 28 after infection to analyze CD4^+^ T cell population by FACS.

### Gene expression analysis

For NanoString analysis, freshly isolated CD4 helper T cells were lysed in RLT buffer (Qiagen). Total RNA was purified using a RNeasy Micro Kit (Qiagen). NanoString reactions were done according to the manufacturer’s instructions, and results were normalized to those of the housekeeping genes included in the “code set.” For QuantiGene gene expression analysis, cultured CD4^+^ T cells were sorted directly into QuantiGene lysis buffer containing proteinase K. QuantiGene multiplex assay was performed according to the manufacturer’s instructions (Thermo Fisher Scientific), and results were normalized to those of the housekeeping genes (TBP, PPIB, and RPLP0).

For human microarray, Tox2 or control virus overexpressed cells were sorted directly into the lysis buffer and RNA was extracted with a Norgen single cell extraction kit for Clariom D human microarray assay. For mice microarray, freshly isolated CD4^+^ T cells form 14 days after H3N2 infection in Tox2^+/+^ Tox2^−/−^ mice spleen were harvested. CXCR5^hi^PD1^hi^ and CXCR5^lo^PD1^lo^ cells were sorted directly into lysis buffer, and RNA was extracted with a Norgen single cell extraction kit for Clariom S mice microarray assay.

### Enzyme-linked immunosorbent assay

For hapten NP-specific enzyme-linked immunosorbent assay (ELISA), NP2-BSA (bovine serum albumin) or NP14-BSA (10 μg/ml; LGC Biosearch Technologies) was coated in PBS. NP2-BSA was used to detect high-affinity Abs, and NP14-BSA was used to detect high- and low-affinity Abs. After blocking with 3% FCS–RPMI 1640, diluted serum samples were added into the plates. Anti-IgG Abs (1:3000 dilution; Rockland), anti-IgG1 Abs (1:500 dilution; A10551; Invitrogen), anti-IgG2b Abs (1:500 dilution; SL257799, Invitrogen), and anti-IgG2c Abs (1:1000 dilution; PA1-29288, Invitrogen) were used for the detection of each isotype. For the virus HA and nuclear protein–specific ELISA, recombinant H3N2 influenza A virus HA protein or recombinant H3N2 influenza A virus nuclear protein (2 μg/ml) was coated on the MaxiSorp ELISA plate (Thermo Fisher Scientific) in KPL coating solution (Seracare/LGC Clinical Diagnostics, Inc.). After blocking with 0.5% milk powder in 0.1% Tween 20 containing PBS, diluted serum samples were added into the plates. Concentrations of each isotypes were analyzed by area under the curve (AUC).

### Statistics

The significance of the difference between groups in the experiments was evaluated by two-tailed paired *t* test or one-way analysis of variance (ANOVA) test. A value of *P* < 0.05 was considered significant.
