# Supplementary material for: Tox2 is required for the maintenance of GC TFH cells and the generation of memory TFH cells
Source: Sci Adv. 2021 Oct 8;7(41):eabj1249. doi: 10.1126/sciadv.abj1249 (PMC8500513; doi:10.1126/sciadv.abj1249)
Supplement: Supplementary file 1 — Figs. S1 to S7 [file sciadv.abj1249_sm.pdf]

Science Advances
AAAS

Supplementary Materials for

Tox2 is required for the maintenance of GC T_{FH} cells and the generation of memory T_{FH} cells

Shu Horiuchi, Hanchih Wu, Wen-Chun Liu, Nathalie Schmitt, Jonathan Provot, Yang Liu, Salah-Eddine Bentebibel, Randy A. Albrecht, Michael Schotsaert, Christian V. Forst, Bin Zhang, Hideki Ueno*

*Corresponding author. Email: ueno.hideki.8e@kyoto-u.ac.jp

Published 8 October 2021, *Sci. Adv.* **7**, eabj1249 (2021)
DOI: 10.1126/sciadv.abj1249

This PDF file includes:

Figs. S1 to S7

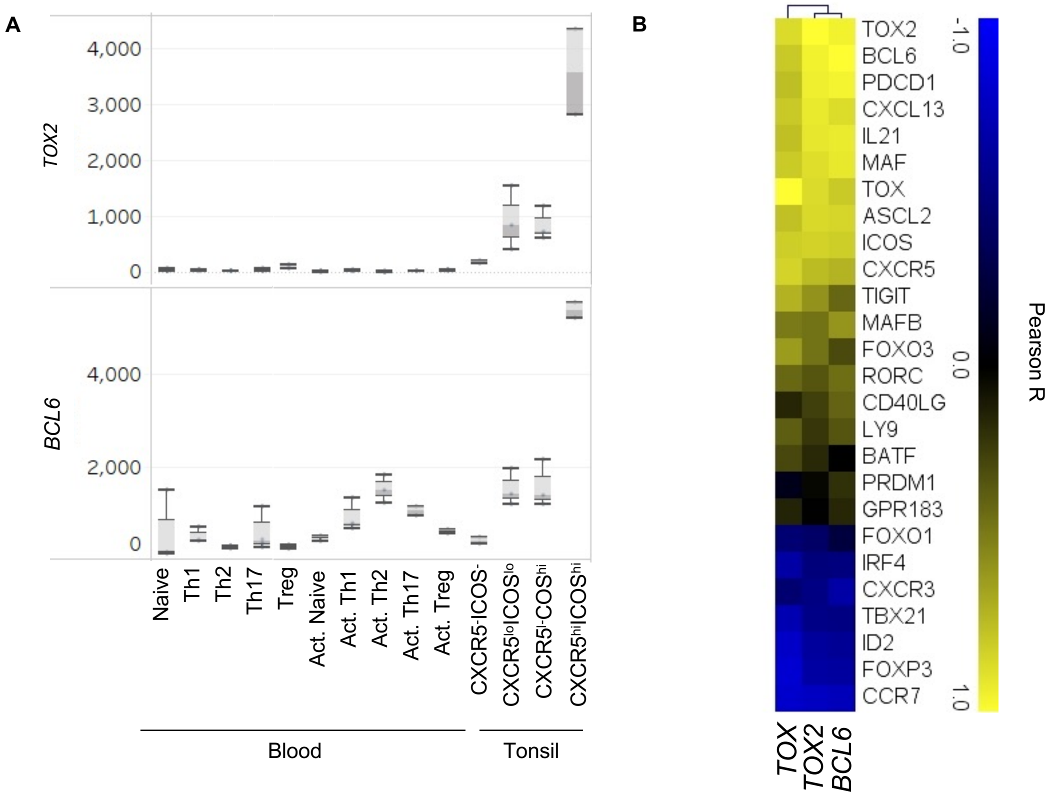

Fig. S1 High Tox2 expression is exclusive in GC Tfh cells

(A) mRNA expression pattern of TOX2 and BCL6 in human tonsillar and blood CD4+ T cell subsets. Human blood CD4+ T cell subsets were sorted as follows: Naïve – CD45RA+CCR7+; Th1 – CD45RA-CXCR3+CCR6-; Th2 – CD45RA-CXCR3-CCR6-CCR4+; Th17 - CD45RA-CXCR3-CCR6+CCR4+; Treg – CD25+CD127-. The sorted cells were activated for 48 h with anti-CD3 and anti-CD28. The expression of TOX2 and BCL6 was analyzed by DNA microarray. N=3.

(B) Correlation between *TOX2, TOX,* and *BCL6* mRNA and Tfh-related molecule mRNA by tonsillar CD4+ T cells after 48 h stimulation with anti-CD3. Indicated by Pearson R values.

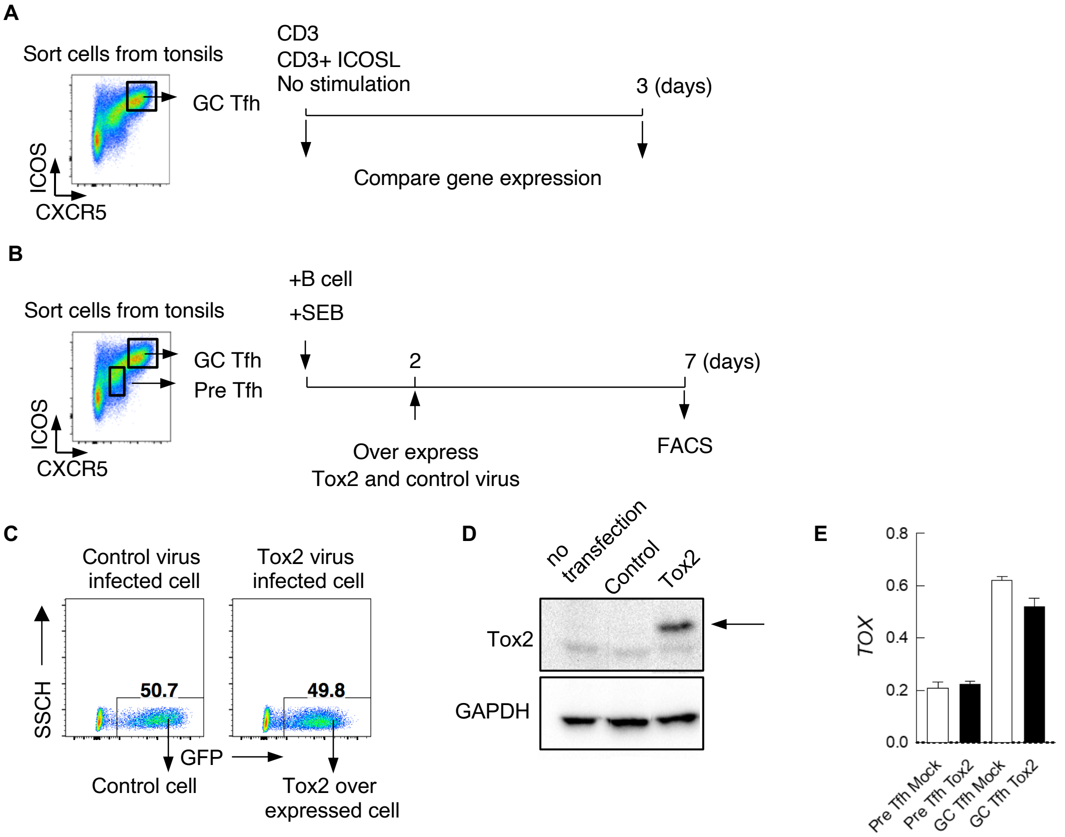

Fig. S2 Human tonsillar Tfh cell culture and Tox2 transfection

(A) Experiment procedure for CD3 and ICOSL stimulated Tonsillar GC Tfh cells. FACS-sorted GC Tfh cells were cultured with CD32-transfected L cells or CD32 and ICOSL-co-transfected L cells in the presence of anti-CD3 for 72 h for transcriptional profiling.

(B) Experiment procedure for Tox2 transfection in Tonsillar GC Tfh and Pre Tfh cells. FACS-sorted GC Tfh or Pre Tfh cells were cultured in the presence of SEB with B cells for 2 days. Cultured cells were transfected with control or Tox2 over expressing HDV and further cultured for additional 5 days for analysis.

(C) Frequency of GFP+ control and Tox2 transfected CD4+ T cells.

(D) Tox2 protein expression in mock or Tox2-transfected cells assessed by WB.

(E) *TOX* gene expression by mock- or Tox2-transfected cells measured by QuantiGene.

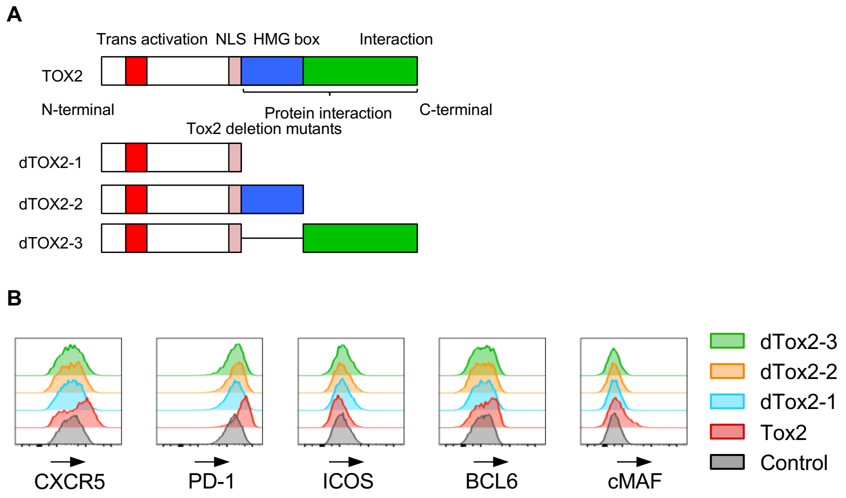

Fig. S3 Tox2 mutants lacking the functional domain do not promote CXCR5 or PD-1 expression by GC Tfh cells

(A) Tox2 mutants were generated by either removing all protein interaction domain (dTOX2-1), interaction domain (dTOX2-2), or HMG box domain(dTOX2-3)

(B) Expression of Tfh cell-associated molecules by control, TOX2, TOX2 deletion mutants lentiviral over-expressed GC Tfh cells. Expression CXCR5, PD-1, and ICOS on cell surface; and Bcl6 and cMaf in cell nucleus was analyzed.

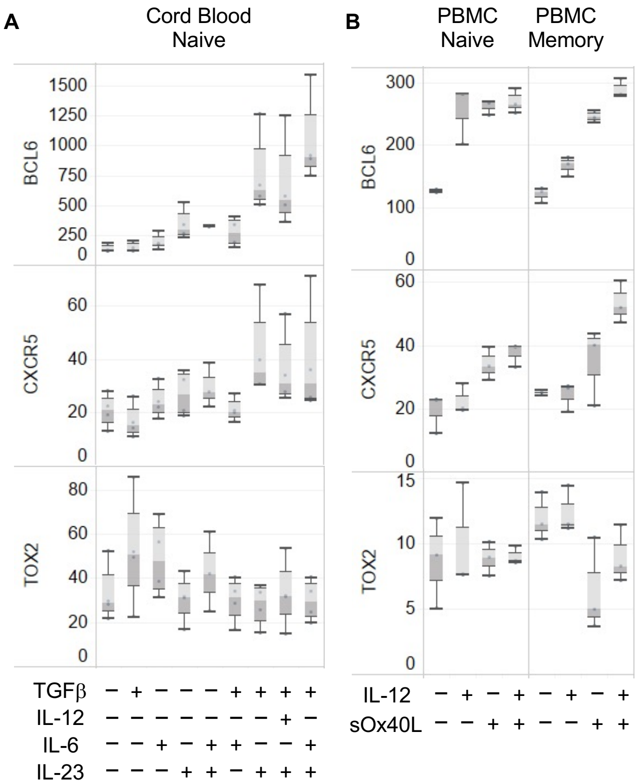

Fig. S4 Tfh-promoting cytokine conditions do not induce TOX2

(A, B) Expression of *BCL6*, *CXCR5, Tox and Tox2* by naive and memory CD4+ T cells obtained from cord blood (H) and blood of human adults (I) cultured for following 4 d stimulation with CD3-CD28 mAbs in the presence of the indicated cytokines (CK) during the last 3 days. *$P < 0.05$ **$P < 0.01$

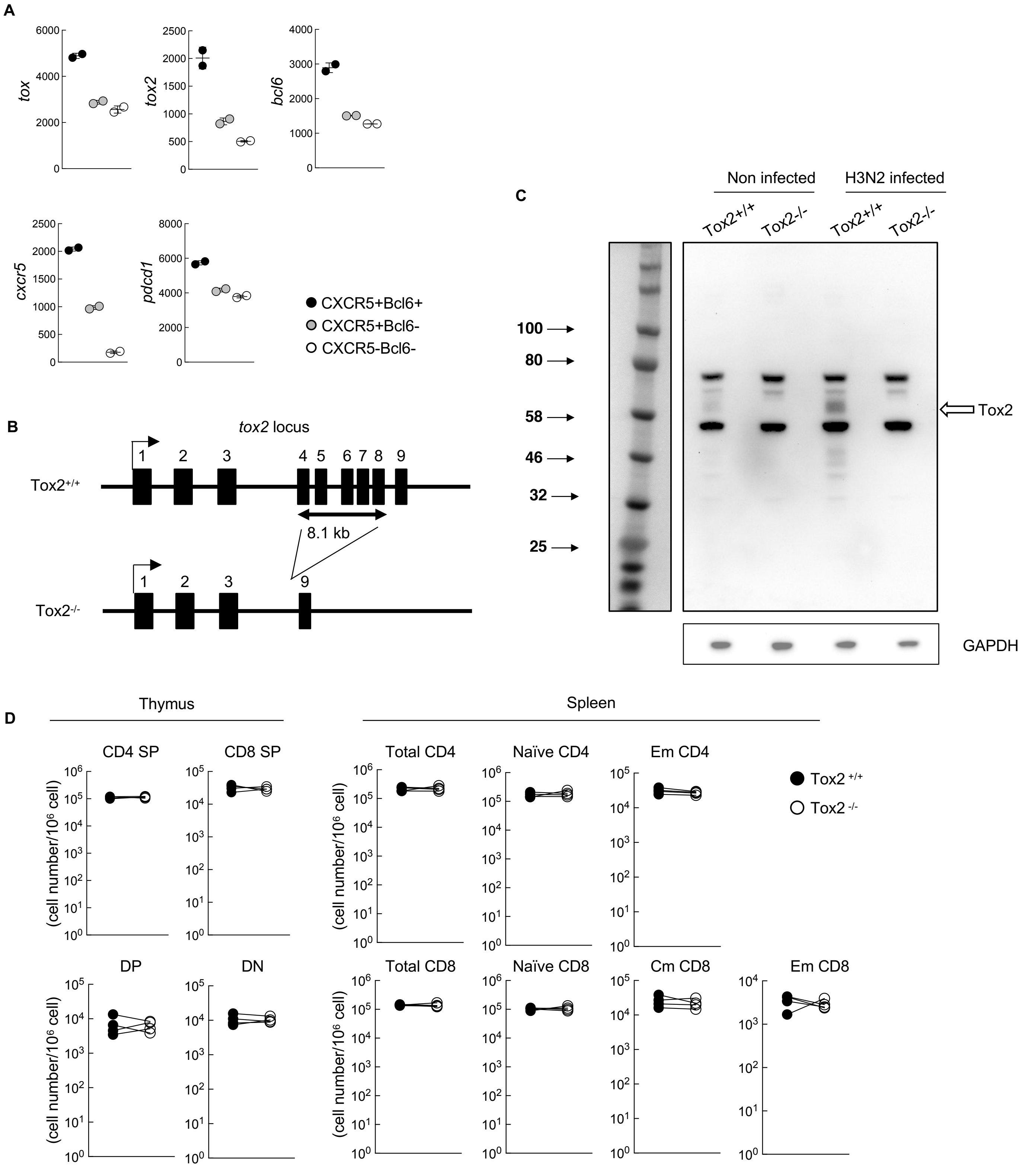

Fig. S5 Generation of Tox2 deficient mice

(A) mRNA expression pattern of tox, tox2, and bcl6 from mice spleen CXCR5-Bcl6-, CXCR5+Bcl6-, CXCR5+ Bcl6+ Tfh cells. Data from GSE40068

(B) Generation of Tox2 knock out mice. Exon 4-8 which codes HMG box domain and interaction domain was removed from the WT allele.

(C) Expression of Tox2 was measured using a mAb to Tox2 by western blot in non infected and d14 H3N2 infected Tox2 +/+ and Tox2 -/- mice Spleen. Whole splenocytes were used and equal amounts of protein were loaded per well to analyze the expression of Tox2.

(D, E) CD4+ T cell and CD8+ T cell number in lymphocytes from Tox2 +/+ and Tox2-/- mice thymus (D) and spleen (E). Each symbol represents the result form an individual mouse paired with littermate.

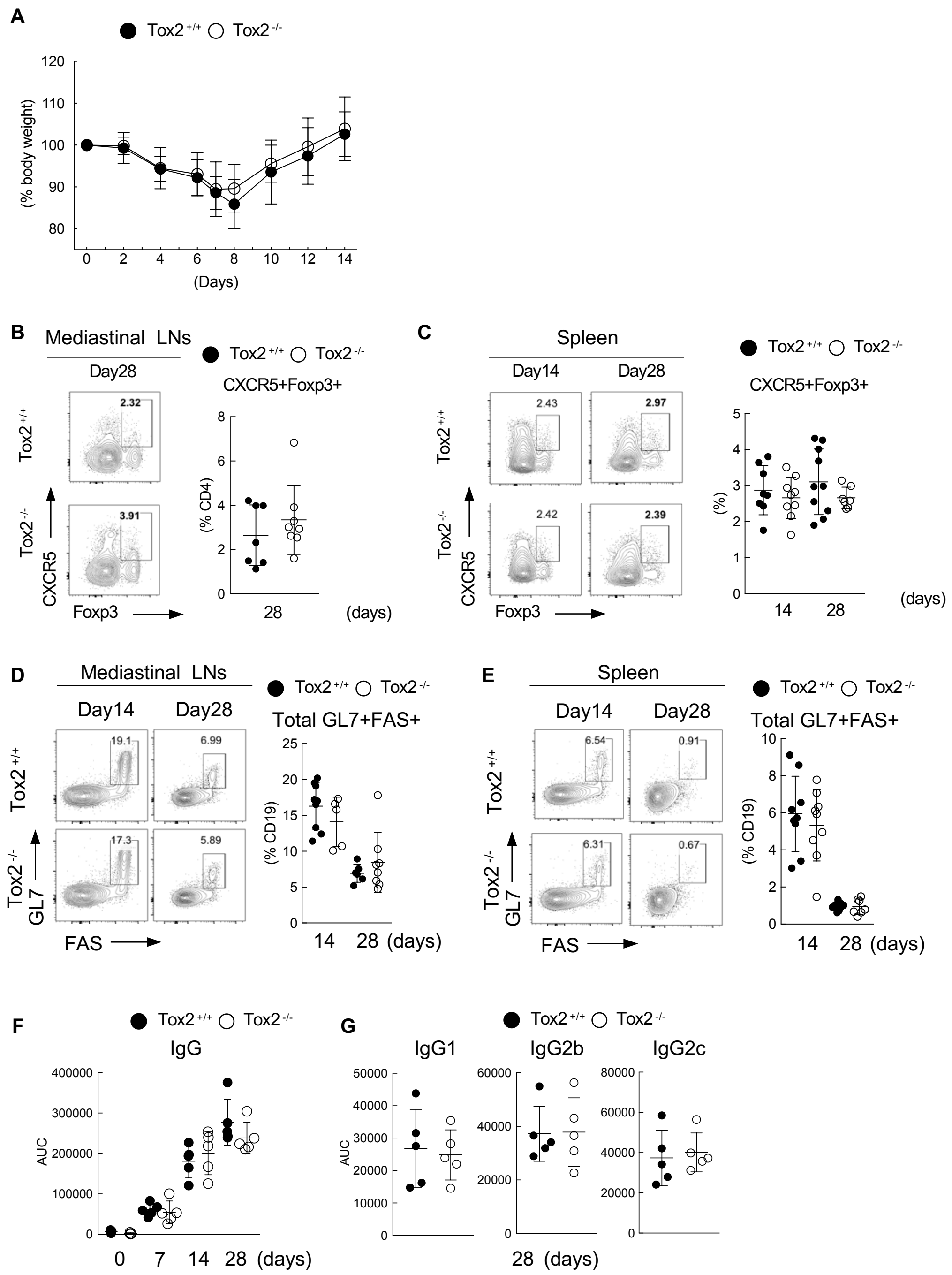

Fig. S6 Tox2 deficiency does not affect GC generation and Tfr

(A) % body weight of Tox2 +/+ and Tox2 -/- mice infected with H3N2 influenza virus.

(B,C) Frequency of Foxp3+CXCR5+ Tfr CD4+ T cells in mediastinal LN (A) and spleen (B) from H3N2 infected C57BL/6 Tox2 +/+ and Tox2 -/- mice at indicated time points.

(D,E) Frequency of total GL7+FAS+ GC B cells in mediastinal LN (C) and spleen (D) at indicated time points.

(F,G) Serum was collected at indicated time points to analyze anti-HA IgG (G), IgG1, IgG2b, IgG2c (H) titer by ELISA. Each symbol represents the result form an individual mouse. 5 to 9 WT and KO mice were used in each experiment. *$P < 0.05$ **$P < 0.01$ ***$P < 0.001$

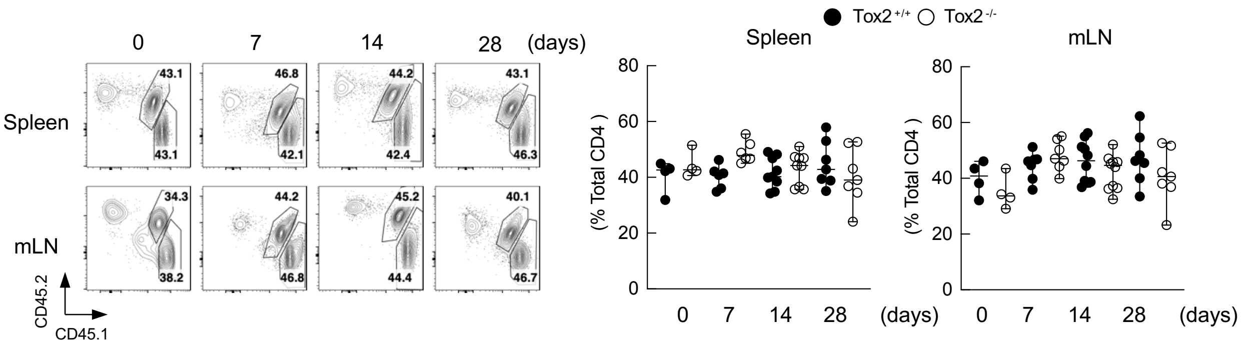

Fig. S7 Frequency of Tox2 +/+ and Tox2 -/- CD4+ T cells were similar in both spleen and mLN before and after infection

Frequency of CD45.1 (Tox2 +/+) and CD45.1/2 (Tox2 -/-) CD4+ T cells in in spleens (B) and in mediastina LNs at indicated time points after H3N2 influenza virus infection. A representative flow data (left) and the data set of four to ten mice are shown (right). Each symbol represents the result form an individual mouse. 4 to 9 BMC were used in each experiment.
